# SINGLE-CELL ANALYSIS OF SKELETAL MUSCLE MACROPHAGES REVEALS AGE-ASSOCIATED FUNCTIONAL SUBPOPULATIONS

**DOI:** 10.1093/geroni/igac059.2672

**Published:** 2022-12-20

**Authors:** Chang-Yi Cui, Linda Krasniewski, Papiya Chakraborty, Krystyna Mazan-Mamczarz, Luigi Ferrucci, Myriam Gorospe

**Affiliations:** National Institute on Aging, Baltimore, Maryland, United States; National Institute on Aging, Baltimore, Maryland, United States; National Institute on Aging, Baltimore, Maryland, United States; National Institute on Aging, Baltimore, Maryland, United States; National Institute on Aging, Baltimore, Maryland, United States; National Institute on Aging, Baltimore, Maryland, United States

## Abstract

Tissue-resident macrophages represent a group of highly responsive innate immune cells that acquire diverse functions by polarizing towards distinct subpopulations. The subpopulations of macrophages that reside in skeletal muscle (SKM) and their changes during aging are poorly characterized. By single-cell transcriptomic analysis with unsupervised clustering, we found eleven distinct macrophage clusters in male mouse SKM with enriched gene expression programs linked to reparative, proinflammatory, phagocytotic, proliferative, and senescence-associated functions. Using a complementary classification, membrane markers LYVE1 and MHCII identified four macrophage subgroups: LYVE1-/MHCIIhi (M1-like, classically activated), LYVE1+/MHCIIlo (M2-like, alternatively activated), and two new subgroups, LYVE1+/MHCIIhi and LYVE1-/MHCIIlo. Notably, one new subgroup, LYVE1+/MHCIIhi, had traits of both M2 and M1 macrophages, while the other new subgroup, LYVE1-/MHCIIlo, displayed strong phagocytotic capacity. Flow cytometric analysis validated the presence of the four macrophage subgroups in SKM, and found that LYVE1- macrophages were more abundant than LYVE1+ macrophages in old SKM. A striking increase in proinflammatory markers (S100a8 and S100a9 mRNAs) and senescence-related markers (Gpnmb and Spp1 mRNAs) was evident in macrophage clusters from older mice. In sum, we have identified dynamically polarized SKM macrophages and propose that specific macrophage subpopulations contribute to the proinflammatory and senescent traits of old SKM.

